# Pentosidine and Bone Properties in Autosomal Dominant Polycystic Kidney Disease

**DOI:** 10.3390/jcm14217577

**Published:** 2025-10-25

**Authors:** Magdalena Jankowska, Abdul Rashid Qureshi, Mathias Haarhaus, Per Magnusson, Alicja Dębska-Ślizień, Peter Barany, Olof Heimburger, Peter Stenvinkel, Bengt Lindholm

**Affiliations:** 1 Department of Nephrology, Transplantology and Internal Medicine, Faculty of Medicine, Medical University of Gdańsk, 80-210 Gdańsk, Poland; 2 Division of Renal Medicine and Baxter Novum, Karolinska Institute, 171 77 Stockholm, Sweden; 3 Department of Clinical Chemistry, and Department of Biomedical and Clinical Sciences, Linköping University, 581 85 Linköping, Sweden

**Keywords:** kidney diseases, advanced glycation end-products, collagen cross-linking, bone strength, bone-specific alkaline phosphatase

## Abstract

**Background/Objectives**: Altered bone metabolism and oxidative stress are features of autosomal dominant polycystic kidney disease (ADPKD). Pentosidine, an advanced glycation end-product and a marker of oxidative stress, has been proposed as an indicator of impaired bone health. This study aimed to evaluate whether pentosidine levels are altered in ADPKD and whether they are associated with bone characteristics in comparison with other chronic kidney disease (CKD) etiologies and healthy individuals. **Methods**: We conducted a cross-sectional analysis of three cohorts comprising 554 adults. Participants were categorized by CKD etiology and stage (G1–G5). ADPKD stages were classified according to the Mayo Imaging Classification (MIC). Plasma pentosidine was analyzed by HPLC and ELISA. Bone material strength index (BMSi) was assessed using a microindentation technique (OsteoProbe^®^). **Results**: Plasma pentosidine was higher in ADPKD compared with other CKD etiologies in CKD stages G1–G4 (*p* = 0.023) and CKD 5D (*p* < 0.0001). Pentosidine was not associated with conventional bone biomarkers. However, in ADPKD individuals with preserved kidney function, higher pentosidine was associated with bone mineral density at the 1/3 radius and with BMSi. **Conclusions**: Pentosidine levels are consistently elevated in ADPKD compared with other CKD etiologies. Associations between pentosidine and measures of cortical bone properties suggest that pentosidine may contribute to skeletal alterations in ADPKD. These findings highlight a novel pathway linking oxidative stress and bone health.

## 1. Introduction

Autosomal dominant polycystic kidney disease (ADPKD) is a genetic and multi-systemic disease caused by pathological variants of genes encoding polycystins [1]. The most important consequence of the deficiency of polycystins is the formation and growth of numerous kidney cysts, leading to progressive decline of kidney function. Ubiquitously expressed in many human tissues, polycystins have functions beyond their role in kidney tubules [2]. Bone involvement represents a relatively recently recognized extra-renal manifestation of ADPKD. While bone disease is a recognized feature of chronic kidney disease (CKD), patients with ADPKD appear to display a distinct skeletal phenotype characterized by relatively preserved bone mass but altered bone quality [3]. A decreased dosage of polycystins in bone results in a low bone turnover [4], preserved cortical bone mineral density (BMD) [5], increased circulating sclerostin [6], together with decreased total alkaline phosphatase (ALP) [7], as observed in ADPKD patients [3,8,9,10]. Interestingly, the systemic course of ADPKD manifests not only as cyst formation but also as low-grade inflammation and oxidative stress [11]. Both are present from the earliest stages of ADPKD [12,13,14].

The altered bone phenotype in ADPKD may have important clinical implications, as highlighted in a recent analysis of fracture rates among individuals from the US Renal Data System across causes of CKD [15]. The fracture rate in ADPKD was the second highest (31 events per 1000 persons per year), exceeded only by that observed in diabetes [15]. However, analysis of the time of the first fracture after dialysis initiation revealed that the fracture risk disproportionately increases in the second and subsequent years, while remaining low at the initiation of kidney replacement therapy [15]. This intriguing finding, together with evidence linking oxidative stress to bone pathology, led us to consider the possibility that compromised bone health in ADPKD may, in part, be related to oxidative stress and the accumulation of advanced glycation end-products (AGEs). With reduced bone turnover, collagen is renewed more slowly, allowing AGEs to accumulate within the bone matrix [16]. This mechanism, also seen with aging, supports our hypothesis that low bone turnover in ADPKD may enhance skeletal AGE accumulation.

Recent studies have highlighted the role of oxidative stress and AGEs in the pathophysiology of CKD and its skeletal complications. AGEs impair osteoblast and osteoclast differentiation and proliferation, and promote apoptosis of mesenchymal stem cells, thereby compromising bone remodeling capacity. [17]. Accumulation of AGEs in bone collagen impacts bone quality and strength in a mechanism of non-enzymatic formation of cross-links within collagen fibers [18]. Among markers of oxidative stress and protein glycation, pentosidine emerges as a factor associated with deteriorated bone quality and risk of fracture [18,19,20,21,22]. Serum pentosidine is a convenient biomarker in investigating bone impairment in oxidative stress, because it is a reliable indicator of its concentration in cortical bone [16,23]. Pentosidine is one of the most intensively studied AGEs, formed through non-enzymatic glycation and oxidation of collagen. Plasma pentosidine levels are markedly elevated in CKD and associated with low GFR, malnutrition and inflammation [24,25]. Moreover, pentosidine has been identified as an independent predictor of mortality in CKD patients, reflecting its pathophysiological relevance beyond a mere byproduct of carbonyl stress. In this context, the coexistence of low bone turnover and oxidative imbalance in ADPKD may provide a permissive environment for accelerated AGE accumulation, thereby contributing to altered bone quality in this population.

We used the bone material strength index (BMSi), assessed with the OsteoProbe^®^ (Active Life Scientific, Santa Barbara, CA, USA), a minimally invasive microindentation technique for evaluating cortical bone quality in vivo. Traditional measures of bone health, such as BMD, do not fully capture bone material properties that determine mechanical competence and fracture risk. Bone material strength index (BMSi) reflects the quality of the bone matrix and its resistance to microfracture. This technique has been validated and introduced as a method ready for clinical use to assess bone strength in individuals at risk of fracture in various populations, including patients with chronic kidney disease, diabetes, and other metabolic bone disorders [26,27].

Our study aimed to test the hypothesis that pentosidine accumulates and influences bone phenotype throughout the stages of ADPKD progression. Therefore, we measured plasma pentosidine levels, bone and mineral biomarkers, and BMSi in patients with ADPKD in comparison to those with other causes of CKD and healthy individuals.

## 2. Materials and Methods

**Figure 1:**
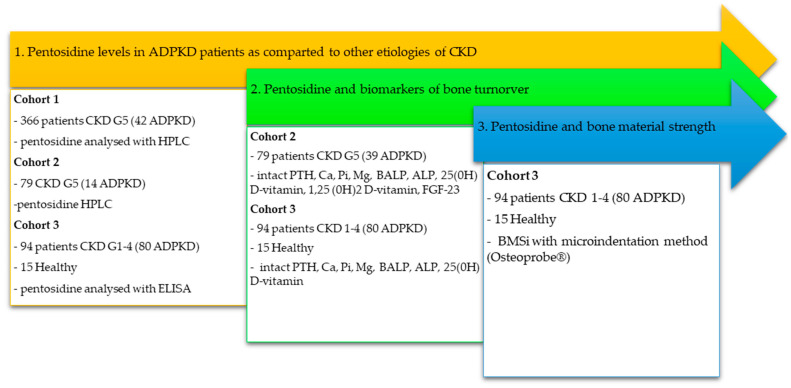
Flowchart summarizing patient cohorts, group allocation, and analyses performed.

We used cross-sectional data from three independent datasets, comprising 524 adult participants, to explore the following: in Study (1) pentosidine in ADPKD vs. other CKD etiologies (n = 524); in Study (2) pentosidine vs. mineral and bone biomarkers (n = 188); and in Study (3) pentosidine vs. bone properties including dual-energy X-ray absorptiometry (DXA) and BMSi (n =109). The study design and main characteristics of cohorts are summarized in Table 1 and Figure 1.

We investigated pentosidine levels in patients with CKD G5 (ADPKD and other etiologies including diabetes) using data from two projects conducted at the Division of Renal Medicine, Karolinska Institute, Stockholm, Sweden.

Cohort 1 (Study 1): The Swedish dataset referred to as Cohort 1 encompasses individuals with CKD G5 enrolled between 1994 and 2016 [28]. In Study 1, we used data from 366 individuals (median age 54 years, 60% men) with available measurements of pentosidine. The causes of CKD were: ADPKD (n = 42), DM (n = 109), chronic primary glomerulonephritis (CGN; n = 92), hypertension/renovascular disease (HT/RVD; n = 75), and other or unknown (n = 48).

Cohort 2 (Study 2): The Swedish dataset, referred to as Cohort 2, included 79 individuals with CKD G5D scheduled for living donor kidney transplantation (between 2001 and 2016). Participants who had assessed pentosidine in plasma (n = 79, median age 45, 41% men) were included in the present study, see Table 1. Among them, 14 were diagnosed with ADPKD, 30 with CGN, 7 with DM, or other primary diseases (n = 28). The median time on preceding dialysis treatment was 1 year. This dataset comprised an assessment of mineral and bone biomarkers, including alkaline phosphatase (ALP) and bone-specific alkaline phosphatase (BALP). This allowed us to analyze associations between pentosidine and bone biomarkers in Study 2.

Cohort 3 (Studies 1, 2 and 3): Cohort 3 consisted of participants in a project which investigated the mineral and bone phenotype of ADPKD patients with preserved renal function, conducted at the Department of Nephrology, Transplantology, and Internal Medicine at the Medical University of Gdansk, Poland. The project excluded participants with diabetes mellitus or inflammation as comorbidities. Also, individuals with contraindications to magnetic resonance imaging (MRI) or local anesthesia (required for BMSi measurement) were excluded. Altogether 109 participants (median age 45 years, 53% men) had accessible measurements of pentosidine and were included in this study. Among these, 94 patients had CKD G1–G4, with 80 (84%) having underlying ADPKD. The remaining participants were diagnosed with IgA nephropathy or other/unknown primary diseases (n = 15). The control group consisted of 14 healthy individuals.

The clinical, demographic and biochemical characteristics of Cohorts 1, 2 and 3 are shown in the tables of the results section. ADPKD was diagnosed clinically according to positive family history and ultrasound criteria, Ravine and Pei.

### 2.1. Materials

After an overnight fast (except for patients on hemodialysis), plasma samples were collected, centrifuged within 30 min of collection, aliquoted, and stored at −70 °C until analysis if not analyzed immediately. Each sample underwent only one freeze–thaw cycle.

Plasma pentosidine was analyzed using two methods as described previously [26,29]: (1) reverse-phase high-performance liquid chromatography (HPLC) in Cohort 1 and Cohort 2 (results expressed as pmol/mg of albumin), and (2) ELISA commercial kit (ABclonal Biotech Co., Woburn, MA, USA) in Cohort 3 (results expressed as ng/mL). Pentosidine was analyzed by HPLC in Cohorts 1 and 2, and by ELISA in Cohort 3. Each method was internally calibrated using reference standards; cross-validation was not required, as between-cohort comparisons were not performed directly.

Serum samples of creatinine, albumin, calcium, phosphate, magnesium, intact parathyroid hormone (PTH), vitamin D, high-sensitivity CRP, ALP and BALP were measured by routine methods at the Department of Laboratory Medicine, Karolinska University Hospital at Huddinge (Cohort 1 and Cohort 2) or the Central Clinical Laboratory, University Clinical Center, Gdańsk (Cohort 3). Estimated GFR (eGFR) was assessed in all patients using the Chronic Kidney Disease Epidemiology Collaboration (CKD-EPI) formula [30]. Stages of CKD relevant for this project are defined as follows: G1—eGFR > 90 mL/min/1.73 m^2^; G2—GFR 60–89 mL/min/1.73 m^2^; G3—30–59 mL/min/1.73 m^2^; G4—15—29 mL/min/1.73 m^2^, and G5—GFR < 15 mL/min/1.73 m^2^.

### 2.2. Imaging and Mayo Clinic Imaging Classification

The stages of ADPKD in Cohort 3 were classified using the Mayo Clinic Imaging (MIC) criteria [31]. In the initial step, an ultrasound evaluation of the kidneys was conducted. Patients with Class 2 disease (characterized by the presence of focal, unilateral, asymmetric, or atrophic changes) were excluded from the study, as prior research indicated that disease progression is highly unpredictable in this group [31]. Class 1 patients were further subdivided into five subclasses based on age and height-adjusted total kidney volume (HtTKV), as assessed by abdominal MRI. Based on these parameters, Class 1 subgroups A, B, C, D, and E were determined, with the risk for GFR deterioration assumed to increase progressively from Class 1A to Class 1E.

### 2.3. Bone Mineral Density and Trabecular Score

Measurements of BMD were performed in Cohort 3 by DXA using a Hologic Discovery densitometer (Hologic QDR-4500A, Hologic, Marlborough, MA, USA) at the whole body, femoral neck (FN), lumbar spine (LS) and 1/3 distal forearm (FA). All measurements were available for 75 ADPKD patients. All scans were performed and interpreted following the International Society of Clinical Densitometry guidelines [32]. Results were expressed as absolute BMD (g/cm^2^). DXA images underwent trabecular bone score (TBS) measurement [iNsight software version 3.0.2 (Medimaps, Merignac, France)].

### 2.4. Bone Material Strength Measurement

Bone material strength (BMS) was assessed in Cohort 3 using the microindentation method with OsteoProbe^®^ equipment (Active Life Scientific, Santa Barbara, CA, USA). After administering local anesthesia, the handheld OsteoProbe^®^ was inserted into the skin at the midshaft of the right tibia (mean distance between the distal apex of the patella and the medial malleolus) until it reached the bone surface, where indentation occurred upon activation of the instrument. The equipment’s software collected a mean of at least ten measurements from the same skin puncture site. Following this, 10 additional measurements were performed on a polymethylmethacrylate (PMMA) plastic calibration standard phantom. BMSi was calculated as 100 times the ratio of the probe’s distance to indent a PMMA standard to the indentation distance measured in bone.

### 2.5. Statistical Analyses

Values are expressed as median (25th–75th percentile) or percentages, as appropriate. Statistical significance was set at the level of *p* < 0.05. Groups were compared using the nonparametric ANOVA, Kruskal–Wallis, and χ^2^ test. The *p*-values were not adjusted for multiple testing and are therefore considered descriptive. Univariate Spearman’s rank correlation was used to determine the association between pentosidine and other variables. We selected variables for the multiple linear regression analysis, which showed significant univariate associations. The results of multivariate linear regression analysis are shown as standardized β regression coefficients. Statistical analyses were performed using statistical software SAS version 9.4 (SAS Campus Drive, Cary, NC, USA).

## 3. Results

### 3.1. Study 1: Pentosidine Plasma Levels in ADPKD vs. Other CKD Etiologies

#### 3.1.1. Pentosidine in CKD G5

In Cohort 1 (Table 2), median plasma pentosidine levels were significantly higher in ADPKD compared with all other CKD etiologies (*p* < 0.001). The next highest levels were observed in HT/RVD, followed by DM.

In CKD G5, increased pentosidine in plasma might have reflected the loss of kidney function. However, the multiple linear regression in the entire cohort (N = 336), including residual kidney function, CRP, age, and sex, identified the diagnosis of ADPKD as the strongest determinant of pentosidine. Full results are shown in Table S1 (Supplementary Materials).

#### 3.1.2. Pentosidine in CKD G5D

In Cohort 2, median plasma pentosidine was 737 (543–1067) pmol/mL. Levels were higher, although not significantly, in ADPKD compared with other CKD etiologies (Table 3). Demographic and clinical characteristics were similar, with no significant differences in BMI, albumin, hsCRP, or other laboratory parameters. Pentosidine indexed to albumin was also higher in ADPKD, but without statistical significance.

#### 3.1.3. Pentosidine in CKD G1–G4 and Healthy Individuals

In Cohort 3, pentosidine levels were highest in ADPKD (CKD G1–G4) compared with healthy controls and patients with other CKD etiologies, excluding DM (Table 4). Expressed per mg of albumin, pentosidine was again highest in ADPKD, intermediate in CKD, and lowest in healthy individuals.

**Figure 2:**
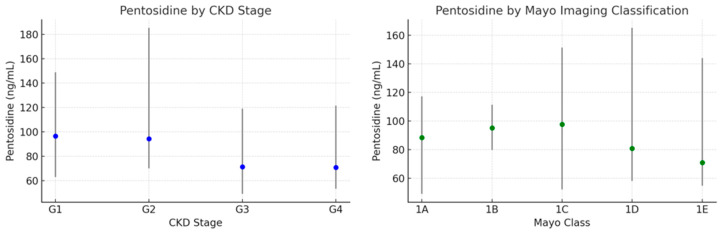
Plasma pentosidine in ADPKD patients from Cohort 3 (N = 80) across stages of chronic kidney disease (CKD) and Mayo Imaging Classification. Data presented as the median (25th–75th percentiles).

Across CKD stages (G1–G4) and MIC classes (1A–1E) in 80 ADPKD patients, pentosidine levels did not differ significantly (Figure 2).

### 3.2. Study 2: Pentosidine and Parameters of Mineral and Bone Metabolism

Key parameters for Cohorts 2 and 3 are presented in Table 5. In CKD G1–G4, serum calcium, phosphate, magnesium, intact PTH, 25(OH)D, ALP, and BALP were similar between ADPKD and other CKD etiologies. In CKD G5D, ADPKD was characterized by higher serum calcium and lower BALP, with no differences in phosphate, PTH, 25(OH)D, or ALP. Persistently lower BALP in ADPKD is consistent with reduced bone turnover reported in previous studies [4].

**Figure 3:**
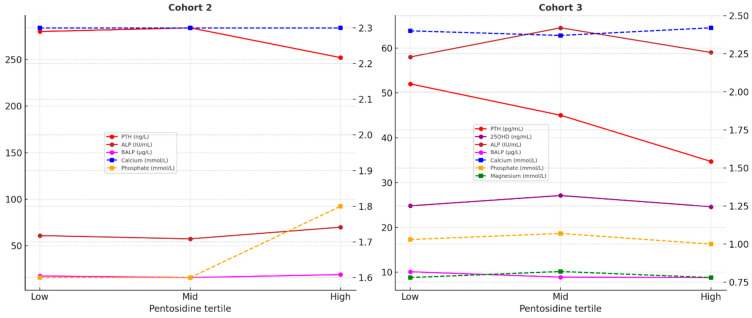
Mineral and bone metabolism parameters across pentosidine tertiles in two independent ADPKD cohorts. Median calcium, phosphate, parathyroid hormone (PTH), alkaline phosphatase (ALP), and bone-specific alkaline phosphatase (BALP) values are shown for low, middle, and high pentosidine tertiles. Cohort 2 N = 79: CKD stage 5 (HPLC). Cohort 3, N = 109: CKD stages 1–4 and healthy controls (ELISA). Separate y-axes were used for low- and high-range parameters.

Analysis of mineral and bone markers across pentosidine tertiles in both cohorts showed no significant differences (Figure 3). Full results in separate cohorts are presented in Supplementary Materials (Tables S2 and S3).

### 3.3. Study 3: Pentosidine, Bone Material Strength Index, and DXA-Derived Bone Phenotype

**Figure 4:**
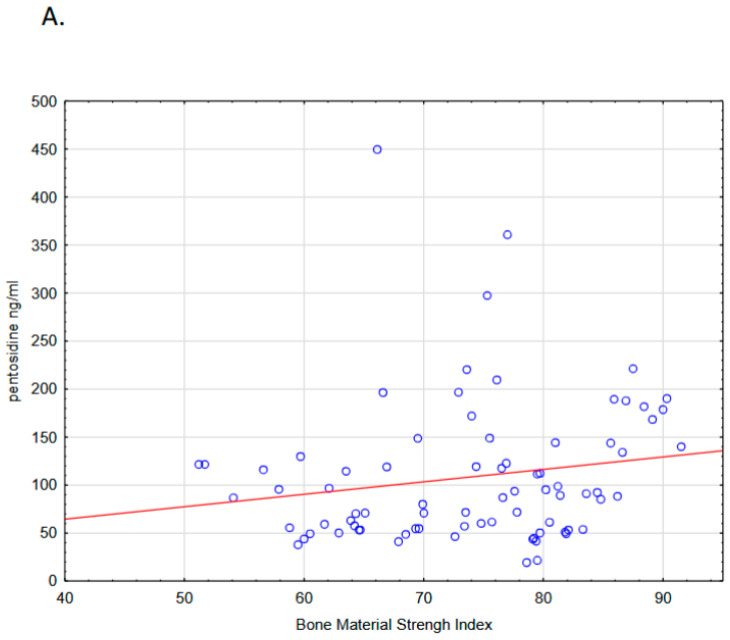

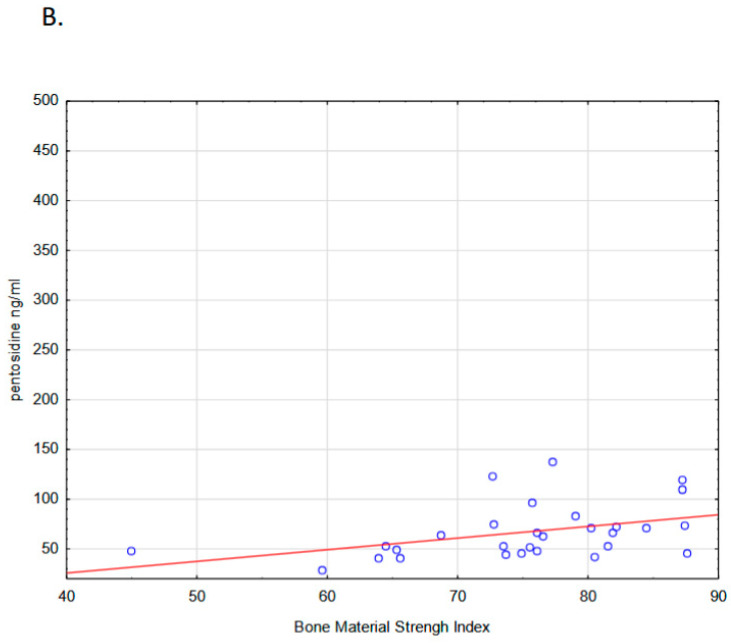
Scatter plots showing the association between plasma pentosidine and bone material strength index (BMSi) in patients with ADPKD (**A**) (N =80; Spearman’s ρ = 0.247; *p* < 0.05) and in other CKD etiologies and healthy controls (**B**) (N = 29; Spearman’s ρ = 0.443; *p* < 0.05) in Cohort 3. The red line represents the linear regression fit for visualization.

In Cohort 3, areal BMD at the whole body, LS, FN, and 1/3 FA, and TBS did not differ significantly between ADPKD and non-ADPKD (Supplementary materials: Table S4). TBS showed no consistent association with pentosidine. In ADPKD, a weak positive correlation with cortical-rich 1/3 FA BMD was seen (rho = 0.258; *p* < 0.05), consistent with the direction of associations for other sites. Interestingly, plasma pentosidine showed a weak but positive association with BMSi in ADPKD patients and non-ADPKD patients, an observation that contrasts with previous reports in other conditions (Figure 4). However, this trend is consistent with the elevated fracture risk discussed in the following section.

## 4. Discussion

We demonstrate for the first time that plasma pentosidine levels are consistently elevated in patients with ADPKD compared with other CKD etiologies. This finding was replicated across three independent cohorts, using two distinct measurement techniques, and was observed at all stages of the disease (CKD G1–G5). These consistent differences suggest that elevated pentosidine is a characteristic feature of ADPKD.

Pentosidine, owing to its low molecular mass (379 Da), depends largely on kidney clearance [33]. In patients with preserved kidney function, additional mechanisms of pentosidine handling may involve cellular metabolism rather than excretion pathways. A plausible explanation for the strikingly increased pentosidine is metabolic reprogramming in ADPKD, affecting glucose metabolism and oxidative stress. The glycolytic switch produces energy mainly through aerobic glycolysis (Warburg effect) [34], often increasing reactive oxygen species and biomarkers of oxidative stress.

We assessed pentosidine levels across ADPKD severity stages, defined by eGFR and HtTKV (MIC), and found no association with either measure. This may reflect limited group sizes, and, as no prior studies have examined pentosidine by CKD grade, our findings could not be confirmed. In our cohort, pentosidine was lower in CKD G5D than in CKD G5. These two patient groups, both from the same Stockholm center, differed in that CKD G5D patients were generally younger and healthier as transplant candidates. Renal replacement therapy likely further contributed to pentosidine reduction in this group [35].

An increase in circulating pentosidine in ADPKD may contribute to fracture risk [23]. Bone material properties are determined in part by the organic matrix, which accounts for up to 45% of cortical bone [36]. Accumulation of AGEs contributes to age-related deterioration of this matrix. Pentosidine, which forms non-enzymatic cross-links between the arginine and lysine of bone collagen, is often found to correlate with the mechanical properties of bone [23]. Cross-links are believed to over-stabilize the collagen and result in a reduction in bone plasticity [37].

Mineral bone turnover can indirectly affect the bone organic matrix. Nevertheless, the relationship between pentosidine and bone turnover remains controversial. We did not observe an association between pentosidine and mineral or bone biomarkers, which goes in line with findings in menopausal women [38] or healthy children [39]. In contrast, a study in 85 hemodialysis patients suggested an association between elevated pentosidine and low PTH [40]. Limited histometric data also indicate a relation between increased pentosidine and low bone turnover [41], a phenotype that characterizes individuals with ADPKD.

In line with our hypothesis, this study demonstrated an association between plasma pentosidine and BMSi. Interestingly, this positive association was unexpected, as previous studies in type 2 diabetes have reported an inverse relationship between pentosidine and BMSi [42]. However, the largest study to date linking BMSi with fracture risk, conducted in 647 elderly women, showed a strong positive association [43]. Collagen cross-links are crucial for the stability of collagen fibers, and cross-linking induced by increased pentosidine would be expected to influence BMSi. Indeed, the enzymatic cross-link pyridinoline has been positively associated with BMSi [44]. Our results are consistent with these observations in CKD and healthy individuals, although pentosidine concentrations in these groups are considerably lower than in ADPKD; hence, the clinical implications may differ.

Our study has several limitations. Its cross-sectional design, use of independent patient cohorts, and application of two different methods for pentosidine measurement may limit direct comparability. The distribution of CKD etiologies, including ADPKD, was unbalanced, and the number of ADPKD patients was relatively small after stratification by CKD stage and MIC class. Finally, as circulating pentosidine is predominantly protein-bound, methodological differences in sample processing could influence its quantification. Additionally, bone turnover markers and molecular indicators related to oxidative stress and pentosidine metabolism were not assessed, which limits the interpretation of bone metabolism dynamics and the underlying mechanisms. Future studies should address these pathways in larger and longitudinal settings. Because ADPKD is a rare disease, extending the cohorts requires multicenter efforts.

Despite these limitations, our study has several strengths. We replicated our findings in three independent cohorts, using two different methods for pentosidine determination, with consistent results across analyses. Importantly, this is the first study to assess pentosidine with BMSi in ADPKD, providing novel insights into cortical bone quality in this population. While larger ADPKD cohorts have been reported in interventional trials, our combined sample of 136 individuals represents one of the largest studied to date in the context of bone research in ADPKD. The integration of biochemical, imaging-based, and microindentation-derived bone measures offers a unique, multidimensional perspective on skeletal involvement in this rare disease.

## 5. Conclusions

In three independent cohorts, plasma pentosidine levels were consistently higher in ADPKD than in other CKD etiologies, across all CKD stages. Pentosidine was not associated with mineral or bone turnover markers but correlated with BMSi, suggesting a relationship with cortical bone mechanical properties. These findings add to current knowledge on the interplay between oxidative stress and bone phenotype and support the potential value of BMSi for skeletal assessment.

## Figures and Tables

**Table 1:** Overview of cohorts and the study design. Continuous variables are presented as median (25–75 percentile).

	Cohort 1 N = 336	Cohort 2 N = 79	Cohort 3 N = 109
Study 1: Pentosidine in ADPKD vs. Other etiologies of CKD	+	+	+
Study 2: Pentosidine and bone biomarkers	-	+	+
Study 3: Pentosidine and measures of bone properties (DXA and BMSi)	-	-	+
Country	Sweden	Sweden	Poland
Age, years	54 (38–63)	45 (31–57)	45 (34–49)
Men (%)	60	41	53
CKD G5 (n)	366	79	0
CKD G1-4 (n)	0	0	94
Healthy (n)	0	0	15
ADPKD (n)	42	14	80
DM (n)	109	7	0
BMI (kg/m^2^)	24.0 (23–26)	23.9 (21.6–25.9)	25.0 (23.2–28.7)
Pentosidine -method of measurement	HPLC	HPLC	ELISA
Bone biomarkers: iPTH, Ca, Pi, Mg, BALP, ALP, 25(0H) D-vitamin	-	+	+
Dual-energy X-ray absorptiometry (DXA)	-	-	+
Bone material strength (BMSi) OsteoProbe^®^	-	-	+

ADPKD, autosomal dominant polycystic kidney disease; ALP, alkaline phosphatase; BALP, bone-specific alkaline phosphatase; BMI, body mass index; Ca, calcium; CKD, chronic kidney disease; DM, diabetes mellitus; Mg, magnesium; Pi, phosphate; iPTH, intact parathormone.

**Table 2:** Demographic data and clinical characteristics of 366 patients from Cohort 1 according to the etiology of chronic kidney disease (CKD). Continuous variables are presented as the median (25th–75th percentiles).

	ADPKD N = 42	DM N = 109	GN N = 92	HT/RVD N = 75	Other/UKN N = 48	*p*-Value
Age (years)	52.0 (48.0–59.0)	58.0 (48.0–65.0)	49.0 (38.5–59.5)	59.0 (48.0–65.0)	57.5 (42.0–65.0)	<0.001
Men, n (%)	20 (47.6%)	74 (67.9%)	54 (58.7%)	49 (65.3%)	26 (54.2%)	0.13
DM, n (%)	0 (0.0%)	109 (100%)	2 (2.2%)	3 (4.0%)	0 (0.0%)	<0.001
BMI (kg/m^2^)	24.7 (22.1–27.7)	25.5 (22.2–29.4)	24.7 (21.6–27.2)	23.5 (21.7–26.4)	22.5 (19.9–25.2)	<0.001
Albumin (g/L)	35.0 (34.0–39.0)	32.0 (28.0–35.0)	34.5 (31.0–38.0)	33.0 (30.0–37.0)	34.0 (29.5–39.5)	<0.001
hsCRP (mg/L)	3.5 (1.3–11.4)	6.3(2.2–15.0)	4.0 (1.6–10.2)	6.8 (2.7–20.2)	7.9 (1.1–17.0)	0.099
Pentosidine (pmol/mL)	1361 (1218–1797)	972 (616–1470)	933 (658–1359)	1282 (782–1532)	741 (654–1133)	<0.001
Pentosidine/albumin (pmol/mg)	39.8 (24.6–52.6)	34.37 (21.3–44.3)	26.2 (19.9–43.5)	37.9 (31.4–53.0)	23.2 (18.2–32.9)	0.009

BMI, body mass index; DM, diabetes mellitus; hsCRP, high-sensitivity C-reactive protein.

**Table 3:** Pentosidine and demographic and clinical characteristics of Cohort 2 (n = 79). Continuous variables are presented as the median (25th–75th percentiles). Comparisons are made between ADPKD G5D and Non-ADPKD CKD G5D individuals.

	ADPKD N = 14	Non-ADPKD N = 65	*p*
Age, years	50	44	<0.001
Males, %	58	63	0.153
BMI, kg/m^2^	24 (23–26)	24 (22–26)	0.631
Albumin, g/L	37 (33–39)	36 (34–39)	0.962
hsCRP, mg/dL	1.1 (0.6–5.3)	0.82 (0.4–2.2)	0.196
Pentosidine (pmol/mL)	861 (652–2164)	728 (536–1061)	0.056
Pentosidine, pmol/mg of albumin	23.27 (16.72–65.58)	20.22 (13.74–31.21)	0.063

BMI, body mass index; hsCRP, high-sensitivity C-reactive protein.

**Table 4:** Pentosidine and demographic and clinical characteristics of Cohort 3 (n = 109). Continuous variables are presented as the median (25th–75th percentiles).

	ADPKD N= 80	Other CKD N = 14	Healthy N = 15	*p*-Value
Age (years)	43.5 (33.5–49.5)	40.5 (37–42)	42 (30–49)	0.07
Sex (% Males)	58 (53)	8 (57)	5 (33)	0.10
DM	0	0	0	n/a
BMI, kg/m^2^	25.1 (23.3–28.7)	23.8 (22.0–28.7)	25.4 (24.3–28.8)	0.85
eGFR CKD_EPI,_ mL/min/1.73 m^2^	58 (41–101)	40 (33–47)	>90	n/a
Albumin, g/L	42 (40–44)	41 (38–42)	43 (42–44)	0.005
hsCRP, g/L	1.14 (0.58–2.67)	1.03 (0.8–1.57)	0.66 (0.3–1.45)	0.42
Pentosidine, ng/mL	214.2(130.7–327.8)	160.5 (120.7–180.7)	133.9 (110.7–187.5)	0.023
Pentosidine /albumin, ng/g	90.1 (58.4–142.0)	58.2 (51.3–71.5)	65.8 (45.1–83.0)	0.066
BMSi	75.6 (65.6–81.3)	74.9 (72.7–82.2)	77.9 (71.7–81.9)	0.587

BMI, body mass index; BMSi, bone material strength index; DM, diabetes mellitus; e-GFR, glomerular filtration rate; hsCRP, high-sensitivity C-reactive protein.

**Table 5:** Mineral metabolism and bone biomarkers in Study 2: ADPKD and comparator groups (n = 188). Variables are presented as the median (25th–75th percentiles).

	Cohort 2			Cohort 3			
Parameter	ADPKD G5D	CKD 5D	*p*-Value	ADPKD G1–G4	CKD3	Healthy	*p*-Value
Calcium, mmol/L	2.36 (2.28–2.43)	2.27 (2.17–2.41)	0.009	2.37 (2.32–2.47)	2.42 (2.32–2.47)	2.39 (2.32–2.47)	0.729
Phosphate, mmol/L	1.6 (1.5–1.9)	1.7(1.2–2.0)	0.426	1.03 (0.90–1.10)	1.02 ( 0.90–1.16)	1.06 (1.03–1.19)	0.173
Magnesium, mmol/L	0.90 (0.80–1.02)	0.84 (0.76–0.93)	0.089	0.78 (0.74–0.82)	0.78 (0.74–0.82)	0.82 (0.82–0.86)	0.026
Intact PTH, ng/L	177 (107–303)	270 (168–465)	0.097	44.9 (29.2–72)	48.6 (42–98)	30 (20.2–40.8)	0.034
25(OH) D-vitamin, nmol/L	48 (29–66)	34 (26–45)	0.017	25.3 (16.3–32.9)	25.2 (17.5–41.4)	28.9 (22.3–36.4)	0.644
ALP, U/L	62.2 (43.1–67.9)	61.3 (47.1–89.8)	0.345	59 (43–75)	65 (58–75)	61 (54–72)	0.438
BALP, μg/L	11.6 (9.2–18.8)	17.9 (11.6–29.5)	0.011	8.3 (6.6–12)	10.5 (8.8–14.3)	10.2 (8.2–12.9)	0.055

ALP, alkaline phosphatase; BALP, bone-specific alkaline phosphatase; PTH, parathormone.

## Data Availability

The data supporting the findings of this study are available from Karolinska Institute, Stockholm, and the Medical University of Gdansk; however, restrictions apply to their availability, and therefore they are not publicly accessible. Data are, however, available from the authors upon reasonable request and with permission of Karolinska Institute and Medical University of Gdansk.

## Supplementary Materials

The following supporting information can be downloaded at: https://www.mdpi.com/article/10.3390/jcm14217577/s1, Table S1: Predictors of increased levels of pentosidine in plasma of 366 patients with chronic kidney disease (CKD) stage G5 in Cohort 1; Table S2: Selected parameters of mineral and bone metabolism across pentosidine tertiles in Cohort 2.; Table S3: Selected parameters of mineral and bone metabolism across pentosidine tertiles in 109 patients with CKD stage Cohort 3; Table S4: Areal Bone Mineral Density and Trabecular score in ADPKD and non-ADPKD patients in Cohort 3.

## Abbreviations

The following abbreviations are used in this manuscript:

ADPKD	Autosomal dominant polycystic kidney disease
AGEs	Advanced glycation end-products
ALP	Alkaline phosphatase
BALP	Bone-specific alkaline phosphatase
BMD	Bone mineral density
BMI	Body mass index
BMSi	Bone material strength index
Ca	Calcium
CKD	Chronic kidney disease
CGN	Chronic primary glomerulonephritis
hsCRP	High-sensitivity C-reactive protein
DM	Diabetes mellitus
FA	Forearm
FN	Femoral neck
eGFR	Estimated glomerular filtration rate
mGFR	Measured glomerular filtration rate
HPLC	Reverse-phase high-performance liquid chromatography
HT/RVD	Hypertension/renovascular disease
Ht/TKV	Height-adjusted total kidney volume
LS	Lumbar spine
Mg	Magnesium
MIC	Mayo Imaging Classification
Pi	Phosphate
PMMA	Polymethylmethacrylate
PTH	Intact parathormone
TBS	Trabecular bone score
