# Supplementary material for: Pentosidine and Bone Properties in Autosomal Dominant Polycystic Kidney Disease
Source: J Clin Med. 2025 Oct 25;14(21):7577. doi: 10.3390/jcm14217577 (PMC12608610; doi:10.3390/jcm14217577)
Supplement: Supplementary file 1 [file jcm-14-07577-s001.zip › jcm-3929200-supplementary.pdf]

Supplementary Table S1.

Predictors of increased levels of pentosidine in plasma of 366 patients with chronic kidney disease (CKD) stage G5 in Cohort 1.

**Total (n=366, adjusted r^2=0.06)**	**Beta**	**T values**	**P value**
Age, years (1-SD)	0.15	2.75	0.006
Gender, *male versus female*	0.02	0.76	0.91
DM, presence versus absence	0.08	0.39	0.60
eGFR ml/min (1-SD)	0.02	0.40	0.68
hsCRP, mg/L (1-SD)	-0.004	-0.07	0.94
DM versus APKD	-0.36	1.67	0.09
CGN versus ADPKD	-0.21	2.67	0.008
HT/RVD versus ADPKD	-0.14	1.83	0.06
Other versus ADPKD	-0.21	2.93	0.004

DM, diabetes mellitus; SGA, Subjective global assessment of nutritional status; eGFR, estimated glomerular filtration rate; hsCRP, high-sensitivity C-reactive protein.

Supplementary Table S2.

Selected parameters of mineral and bone metabolism across pentosidine tertiles in Cohort 2.

	**All**	**Low pentosidine tertile**	**Middle pentosidine tertile**	**High pentosidine tertile**	**P-value**

	N=79	N=26	N=26	N=27	
Pentosidine pmol/ml	737 (543-1067)	477 (377-543)	736 (690-815)	1245 (1059-1728)	<0.001
Age years	45.0 (31.0-57.0)	39.5 (29.0-50.0)	47.0 (40.0-55.0)	50.0 (31.0-62.0)	0.20
Sex (% men)	32 (41%)	14 (54%)	9 (35%)	9 (33%)	0.24
BMI kg/m^2	23.9 (21.6-25.9)	22.4 (20.9-25.7)	24.6 (22.3-26.2)	23.9 (21.9-26.4)	0.20
Calcium mmol/l	2.3 (2.2-2.4)	2.3 (2.2-2.4)	2.3 (2.2-2.4)	2.3 (2.2-2.5)	0.40
Phosphate mmol/l	1.7 (1.3-2.0)	1.6 (1.2-1.8)	1.6 (1.4-1.9)	1.8 (1.7-2.1)	0.015
PTH ng/l	260 (170-465)	280 (198-522)	284 (176-421)	252 (112-384)	0.30
ALP IU/ml	62.0 (47.0-90.0)	61.0 (47.0-90.0)	57.5 (48.0-84.0)	70.0 (51.0-113.0)	0.47
BALP µg/l	17.3 (11.3-28.7)	17.6 (11.8-26.0)	15.9 (11.1-24.0)	19.1 (10.9-34.2)	0.65

ALP, alkaline phosphatase; BALP, bone specific alkaline phosphatase; BMI, body mass index; PTH, parathormone

Supplementary Table S3.

Selected parameters of mineral and bone metabolism across pentosidine tertiles in 109 patients with CKD stage Cohort 3

	**All** N=109	**Low pentosidine tertile** N=36	**Middle pentosidine tertile** N=36	**High pentosidine tertile** N=37	**P-value**
Pentosidine ng/ml	72 (53-22)	49 (43-53)	72 (65-89)	144 (122-190)	<0.001
Age years	42 (33-49)	43 (34-52)	42 (35-49)	41 (33-48.)	0.65
Sex (%Men)	58 (53.2%)	16 (44.4%)	21 (58.3%)	21 (56.8%)	0.43
BMI (kg/m^2)	25.1 (23.4-28.7)	25.1 (23.2-28.9)	26.6 (24.2-28.8)	24.6 (23.1-27.9)	0.26
Calcium mg/dL	9.6 (9.3-9.9)	9.6 (9.3-9.9)	9.5 (9.2-9.6)	9.7 (9.5-10.0)	0.066

Phosphate mg/dL	3.2 (2.8-3.5)	3.2 (2.9-3.7)	3.3 (2.9-3.5)	3.1 (2.8-3.5)	0.44
Magnesium	1.9 (1.8-2.1)	1.9 (1.8-2.2)	2.0 (1.9-2.0)	1.9 (1.8-2.0)	0.074
PTH pg/ml	43.9 (27.7-69.8)	52.0 (29.6-99.0)	45.0 (26.6-60.3)	34.7 (24.8-56.8)	0.18
25OH D ng/mL	25.4 (17.5-34.4)	24.8 (16.6-34.1)	27.1 (20.8-36.4)	24.6 (14.6-32.7)	0.32
ALP IU/ml	60.0 (46.0-72.0)	58.0 (42.5-77.5)	64.5 (49.5-75.0)	59.0 (49.0-69.0)	0.72
BALP µg/l	9.0 (6.8-12.5)	10.1 (6.8-13.6)	8.9 (6.1-11.9)	8.8 (7.3-12.2)	0.63

ALP, alkaline phosphatase; BALP, bone specific alkaline phosphatase; BMI, body mass index; PTH, parathormone

Supplementary Table S4.

Areal Bone Mineral Density and Trabecular score in ADPKD and non-ADPKD patients in Cohort 3

	**ADPKD (n=75)**	**Non ADPKD (n =29)**	**P value**
TBS	1.41 (1.32 – 1.49)	1.45 (1.38-1.51)	0.071
WB BMD (g/cm2)	1.14 (1.08-1.21)	1.14 (1.06-1.21)	0.791
LS BMD (g/cm2)	1.01 (1.02-1.12)	1.08 (1.02-1.12)	0.138
FN BMD (g/cm2)	0.86 (0.76-0.96)	0.84 (0.78-0.90)	0.103
1/3 FA BMD (g/cm2)	0.74 (0.69-0.81)	0.72 (0.68-0.79)	0.578

FN- femoral neck; LS- lumbar spine; FA- forearm; TBS- trabecular score; WB BMD- whole body bone mineral density;
